# Ten-year trends of antibiotic prescribing in surgery departments of two private sector hospitals in Central India: a prospective observational study

**DOI:** 10.1186/s12889-024-17817-2

**Published:** 2024-01-27

**Authors:** Kristina Skender, Anna Machowska, Shyam Kumar Dhakaita, Cecilia Stålsby Lundborg, Megha Sharma

**Affiliations:** 1 Department of Global Public Health, Health Systems and Policy, Karolinska Institutet, 17177 Stockholm, Sweden; 2 Department of Surgery, Ruxmaniben Deepchand Gardi Medical College, Surasa, Ujjain, 456006 India; 3 Department of Pharmacology, Ruxmaniben Deepchand Gardi Medical College, Surasa, Ujjain, 456006 India

**Keywords:** Antibiotic prescribing trends, AWaRe, Surgery, Private sector hospitals, Teaching hospital, Non-teaching hospital, Antimicrobial resistance, Essential medicines list, Prophylactic antibiotic, India

## Abstract

**Background:**

Inappropriate antibiotic use contributes to the global rise of antibiotic resistance, prominently in low- and middle-income countries, including India. Despite the considerable risk of surgical site infections, there is a lack of antibiotic prescribing guidelines and long-term studies about antibiotic prescribing in surgery departments in India. Therefore, this study aimed to analyse 10 years’ antibiotic prescribing trends at surgery departments in two tertiary-care hospitals in Central India.

**Methods:**

Data was prospectively collected from 2008 to 2017 for surgery inpatients in the teaching (TH-15,016) and the non-teaching hospital (NTH-14,499). Antibiotics were classified based on the World Health Organization (WHO) Access Watch Reserve system and analysed against the diagnoses and adherence to the National List of Essential Medicines India (NLEMI) and the WHO Model List of Essential Medicines (WHOMLEM). Total antibiotic use was calculated by DDD/1000 patient days. Time trends of antibiotic prescribing were analysed by polynomial and linear regressions.

**Results:**

The most common indications for surgery were inguinal hernia (TH-12%) and calculus of the kidney and ureter (NTH-13%). The most prescribed antibiotics were fluoroquinolones (TH-20%) and 3^rd^ generation cephalosporins (NTH-41%), and as antibiotic prophylaxis, norfloxacin (TH-19%) and ceftriaxone (NTH-24%). Access antibiotics were mostly prescribed (57%) in the TH and Watch antibiotics (66%) in the NTH. Culture and susceptibility tests were seldom done (TH-2%; NTH-1%). Adherence to the NLEMI (TH-80%; NTH-69%) was higher than adherence to the WHOMLEM (TH-77%; NTH-66%). Mean DDD/1000 patient days was two times higher in the NTH than in the TH (185 vs 90). Overall antibiotic prescribing significantly increased in the TH (β_1_ =13.7) until 2012, and in the NTH (β_2_ =0.96) until 2014, and after that decreased (TH, β_2_= -0.01; NTH, β_3_= -0.0005). The proportion of Watch antibiotic use significantly increased in both hospitals (TH, β=0.16; NTH, β=0.96).

**Conclusion:**

Total antibiotic use decreased in the last three (NTH) and five years (TH), whereas consumption of Watch antibiotics increased over 10 years in both hospitals. The choice of perioperative antibiotic prophylaxis was often inappropriate and antibiotic prescribing was mostly empirical. The results of this study confirmed the need for antibiotic prescribing guidelines and implementation of antimicrobial stewardship programs.

## Introduction

Antimicrobial resistance (AMR) is a global health threat that contributes to more than five million deaths every year worldwide [1]. AMR is primarily driven by the inappropriate use of antibiotics, which ultimately leads to a decrease in their effectiveness and an increased necessity for frequent use of broad-spectrum antibiotics to treat common infections [1, 2]. Despite rising awareness of the AMR problem, antibiotic consumption is on the rise globally. Especially in the low-and middle-income countries (LMICs), antibiotic consumption continues to increase, due to still high infectious disease burden, insufficient vaccination coverage, rising incomes, lower drug prices, increased access, unregulated prescription and sales, inadequate infection prevention and control measures, lack of diagnostic ability and surveillance, and lack of education and public awareness [2–5]. India is one of the biggest antibiotic consumers in the world [2, 4, 6] and showed the overall greatest increase (103%) in antibiotic consumption between 2000 and 2015 among all LMICs [4]. In 2019, an analysis of aggregate levels of drug resistance showed that India had the worst drug resistance index among 41 countries, resulting in the lowest level of antibiotic effectiveness [7].

In 2017, the World Health Organization (WHO) developed the Access, Watch, and Reserve (AWaRe) Classification to promote rational use of antibiotics, global comparison and support local and national policy development and antibiotic stewardship efforts [1]. Antibiotics are classified into AWaRe groups, based on their AMR potential and preference for use. The WHO has set a target that by 2023 in every country, a minimum of 60% of all prescribed antibiotics should be from the Access group [1]. In India, total consumption of Access antibiotics decreased by 13% from 2011 to 2019, whereas consumption of Reserve antibiotics increased by 247% [2], which indicates rising levels of AMR [4].

Despite widespread antibiotic use and advancements in surgical techniques, surgical site infections (SSIs) remain a significant cause of morbidity, mortality, and increased healthcare costs globally [8–10]. SSIs are the most common healthcare-associated infections that develop due to contaminated instruments or environment at the healthcare facility within 30 days after surgery or within 90 days if an implant is left in place; and can involve skin, tissues under the skin, organs, or implanted material [11–13]. One of the recommended measures to minimize the risks and consequences of SSIs is the administration of systemic antibiotics before or during surgery, i.e., perioperative antibiotic prophylaxis (PAP) [14, 15]. It is estimated that 30-50% of all antimicrobials in hospitals are used for PAP [16], up to 60% of surgical patients receive postoperative antibiotics during hospital stay, and up to 50% are discharged with antibiotics [17]. A significant proportion of antibiotic prescribing in surgery departments is reported to be inappropriate, which consequently contributes to AMR in a vicious cycle [16–18]. The most common drivers of inappropriate antibiotic prescribing are incorrect dose, frequency or duration of therapy, and use of broad-spectrum antibiotics when narrow-spectrum would suffice [19, 20]. Reported reasons for inappropriate antibiotic prescribing in surgery are uncertainty in diagnosis, complex comorbidities, prescriber's lack of experience or training, unfamiliarity with local resistance patterns, lack of laboratory capacity, and mistakes in microbiological results' interpretation [10].

The incidence of SSIs is considerably higher in LMICs than in high-income countries [8, 21]. In India, the risk of acquiring SSI varies largely across the country and between healthcare facilities and ranges between 1.6% and 38% [22]. SSI incidence in the study setting was estimated between 5 and 7.5% in different time periods and departments (general surgery and orthopaedic surgery) [23, 24]. Despite the high SSI risk, there is no national policy nor guidelines for PAP and antibiotic prescribing in surgery, and antibiotic choices are often empirical [25, 26].

Ninety-three percent of all hospitals in India belong to the private sector [27] and it is estimated that up to 90% of total drug consumption occurs in the private sector health facilities [2]. Yet the research on antibiotic prescribing has mostly been conducted in public hospitals. Consequently, leading to a knowledge gap in antibiotic prescribing patterns in the major healthcare sector of India [28–30]. Thus, it is crucial to estimate the actual use of antibiotics before planning and implementing antibiotic stewardship programs in the private hospitals. Previous research conducted in private hospitals in Madhya Pradesh suggested that the most commonly prescribed antibiotics were broad-spectrum and often prescribed empirically without a clear indication [28–31]. The WHO emphasizes on the importance of long-term surveillance data for better reliability in developing and implementing antibiotic prescribing policies and antibiotic stewardship programs. However, there was no research done on long-term antibiotic prescribing trends in surgery departments in private-sector hospitals in Central India. Therefore, the present study aims to analyse and present 10-year antibiotic prescribing patterns, trends, and their appropriateness at group level in surgery departments of two private sector hospitals in Madhya Pradesh.

## Methods

### Study setting

The study was conducted in surgery departments of two tertiary private-sector hospitals: a teaching hospital (TH) and a non-teaching hospital (NTH) in Ujjain district, Madhya Pradesh, India. The TH is associated with Ruxmaniben Deepchand Gardi Medical College located on the outskirts of Ujjain city, with a total of 800 beds and a capacity of 130 beds at the surgery departments, which provided healthcare and medicines free of charge at the time of study. The NTH is a city-based hospital with a total of 400 beds, out of which 36 beds at the surgery department, where patients must pay out-of-pocket for health services and medicines. Both hospitals have functional microbiology laboratories.

### Data collection and management

Data was prospectively collected from 2008 to 2017. All nurses were pre-trained by the same person (MS) for data collection, and the same paper form and same procedure were used in both hospitals to maintain uniformity. Paper forms were designed to collect information about patients' demographic characteristics, clinical diagnoses, hospitalization dates, operation status, prescribed antibiotics, and, if performed, culture and susceptibility tests. Regular monitoring and cross-checking of the data collection forms with the patient files were done by same person (MS) to control the accuracy of the data collection process.

Data was collected for all patients admitted to the surgery departments in both study hospitals (TH-21,339; NTH-20,550). Patients below 15 years (TH-1,911; NTH-451), patients who stayed less than one day in the surgery departments (TH-842; NTH-2000), those who did not have complete information about the diagnosis (TH-242; NTH-0), and those who were not prescribed antibiotics during the hospital stay (TH-3,328; NTH-3,600) were excluded from the analysis. Thus, we ended up with the study population of 15,016 surgical patients in the TH and 14,499 surgical patients in the NTH, who were prescribed antibiotic(s) during hospital stay.

Antibiotic prescriptions were classified based on the WHO Anatomical Therapeutic Chemical (ATC) Classification. For each antibiotic, a Defined Daily Dose (DDD) was given, according to the 2023 ATC/DDD Index by the WHO Collaborating Centre for Drug Statistics Methodology (WHOCC) [32]. Metric DDD/1000 patient days was used as an indicator of total antibiotic consumption. Further, antibiotic prescriptions were checked across the National List of Essential Medicines of India (NLEMI, 2022) [33] and the WHO Model List of Essential Medicines (WHOMLEM, 2023) [34]. Additionally, prescribed antibiotics were categorized based on the AWaRe Classification (2022) [1].

Diagnoses were coded according to the International Statistical Classification of Diseases and Related Health Problems-10th Revision (ICD-10, 2016) [35], which was valid at the time of the study. Prescribed antibiotics were analysed against the ten most common diagnoses in each study hospital. For the most common diagnoses in each hospital, number of operated cases was calculated, and the respective antibiotic prophylaxis was presented. The antibiotic prophylaxis information was obtained from the data about the antibiotics prescribed on the day of surgery. The appropriateness of antibiotic prescription was assessed at group level by: i) analysis of the most prescribed antibiotics against the most common diagnoses, ii) analysis of operation status of the most common diagnoses and the respective antibiotic prophylaxis used in comparison with the guidelines, iii) estimation of the median length of antibiotic therapy, iv) assessment of the frequency of culture and susceptibility testing, v) adherence of prescribed antibiotics to the NLEMI and the WHOMLEM, and vi) assessment of the proportion of AWaRe antibiotics used.

### Statistical analysis

For categorical variables, frequencies and percentages were calculated and compared between the two hospitals by Pearson chi-squared test. For the variables where the number of sample observations was less than five, Fisher’s exact test was used for comparison of frequency and percentage.

#### Time series analysis

Time series analysis was used to assess the trends of antibiotic use over time. Residual analysis and Wald test were conducted to check for the linearity of trends. The trends proved to be quadratic polynomial in the TH and cubic polynomial in the NTH. Heteroskedasticity test was performed to check for the goodness of fit of the regression models, and no heteroskedasticity was found. Additionally, Akaike information criteria (AIC) and Bayesian information criteria (BIC) were calculated to choose the best model. Therefore, polynomial regression was used to evaluate the changes in trends of total antibiotic use over time. The rate of change by month was given by coefficients (β_1_, β_2_, β_3_), defined as the slopes of response over time. Linear model was chosen as a better fit for the trend of Access/Watch/Reserve ratio over time, and thus linear regression was used to analyse the changes in trends, with a coefficient β representing the rate of change by month. *P*-values <0.05 were considered statistically significant. Data was analysed using STATA SE version 17.0 (Stata Corp., College Station, TX, USA).

## Results

In total, the study population consisted of 29,515 surgical patients (TH, 15,016; NTH, 14,499, Table 1). In both hospitals, most patients were male (TH, 78%; NTH, 71%) and below 46 years of age (TH, 53%; NTH, 61%). Median length of hospital stay was longer in the TH (12 days) than in the NTH (4 days). In both hospitals, the majority of the patients were prescribed one antibiotic for five days. Culture and susceptibility tests were rarely performed (TH, 2%; NTH, 1%). More patients were prescribed antibiotics at discharge in the NTH (50%) compared to the TH (14%, Table 1).

**Table 1:** Patients’ characteristics at surgery departments in the teaching and the non-teaching hospital in Ujjain, India

Patients’ characteristics	Teaching hospital	Non-teaching hospital	*P*-value
	***n*= 15,016**	***n*=14,499**	
	**N (%)**	**N (%)**	**χ**^**2**^** test**
Sex			
*Male*	11,676 (78)	10,344 (71)	*<*0.001
*Female*	3,340 (22)	4,155 (29)	*<*0.001
Age			
*15-30*	3,950 (27)	4,666 (32)	*<*0.001
*31-45*	3,933 (26)	4,267 (29)	*<*0.001
*46-60*	3,973 (27)	2,993 (21)	*<*0.001
*>60*	3,034 (20)	2,545 (18)	*<*0.001
*N/A*^a^	126 (0)	28 (0)	*<*0.001
Outcome			
*Discharged*	10,316 (69)	13,330 (92)	*<*0.001
*Shifted to other wards*	161 (1)	728 (5)	*<*0.001
*Absconded*	1,297 (8)	220 (2)	*<*0.001
*Discharged on request*	97 (1)	12 (0)	*<*0.001
*Referred to other hospital*	3,144 (21)	182 (1)	*<*0.001
*Died*^a^	1 (0)	27 (0)	*<*0.001
Culture and susceptibility test	261 (2)	106 (1)	*<*0.001
Antibiotic(s) at discharge	2,062 (14)	7,274 (50)	*<*0.001
Number of antibiotic substances per patient, Median	1	1	
Length of antibiotic treatment in hospital, days, Median	5	5	
Length of hospital stay, days, Median	12	4	

^a^Fisher’s exact test was used instead of the χ2 test for comparison of the sample observations

Table 2 shows that in total 257,205 (TH- 178,712; NTH- 78,493) antibiotic prescriptions were prescribed during the study period. Fluoroquinolones (20%) in the TH and 3^rd^ generation cephalosporins (41%) in the NTH were the most prescribed antibiotic classes, whereas oral metronidazole (10%) in the TH and ceftriaxone (16%) in the NTH were the mostly prescribed antibiotics. Adherence to the NLEMI (TH, 80%; NTH, 69%) was higher than adherence to the WHOMLEM (TH, 77%; NTH, 66%) in both hospitals. Fixed-dose combinations (FDCs) of antibiotics were more frequently prescribed in the NTH (31%) compared to the TH (17%). Most of the prescribed antibiotics belonged to the Access group in the TH (57%) and the Watch group in the NTH (66%, Table 2).

**Table 2:** Antibiotic prescription patterns at surgery departments in the teaching and the non-teaching hospital in Ujjain, India

ATC Codes	Antibiotic prescriptions	Teaching hospital *n*=178,712 N (%)	Non-teaching hospital *n*=78,493 N (%)	*P-*value
J01AA	TETRACYCLINES	10,949 (6)	131 (0)	<0.001
J01AA02	*Doxycycline*	10,949 (6)	131 (0)	*<*0.001
J01CR	*COMBINATIONS OF PENICILLINS, INCLUDING β- LACTAMASE INHIBITOR*	15,227 (9)	9,999 (13)	*<*0.001
J01CR02	*Amoxicillin and β-lactamase inhibitor*	8,150 (5)	4,868 (6)	<0.001
J01CR05	*Piperacillin and β-lactamase inhibitor*	1,592 (1)	4,695 (6)	<0.001
J01DB	*1*^*st*^* GENERATION CEPHALOSPORINS*	3,968 (2)	1,601 (2)	0.004
J01DC	*2*^*nd*^* GENERATION CEPHALOSPORINS*	1,379 (1)	2,750 (4)	<0.001
J01DD	*3*^*rd*^* GENERATION CEPHALOSPORINS*	26,214 (15)	32,117 (41)	<0.001
J01DD01	*Cefotaxime*	17,880 (10)	1,509 (2)	<0.001
J01DD04	*Ceftriaxone*	3,093 (2)	12,555 (16)	<0.001
J01DD62	*Cefoperazone and β-lactamase inhibitor*	712 (0)	5,083 (7)	<0.001
J01DD63	*Ceftriaxone and β-lactamase inhibitor*	3,450 (2)	7,838 (10)	<0.001
J01EE	COMBINATIONS OF SULFONAMIDE AND TRIMETHOPRIM	9,481 (5)	28 (0)	<0.001
J01FF	*LINCOSAMIDES*	9,863 (6)	340 (0)	<0.001
J01GB	*OTHER AMINOGLYCOSIDES*	27,433 (15)	12,391 (16)	0.005
J01GB03	*Gentamicin*	13,768 (8)	1,596 (2)	<0.001
J01GB06	*Amikacin*	13,653 (8)	10,767 (14)	<0.001
J01MA	*FLUOROQUINOLONES*	36,027 (20)	10,632 (14)	<0.001
J01MA01	*Ofloxacin*	167 (0)	3,244 (4)	<0.001
J01MA02	*Ciprofloxacin*	17,157 (10)	1,978 (3)	<0.001
J01MA06	*Norfloxacin*	17,689 (10)	263 (0)	<0.001
J01MA12	*Levofloxacin*	820 (0)	2,829 (4)	<0.001
J01XD	*IMIDAZOLE DERIVATIVES*	16,725 (9)	6,156 (8)	<0.001
J01XD01	*Metronidazole (IV)*	16,725 (9)	6,156 (8)	<0.001
P01AB	*NITROIMIDAZOLE DERIVATIVES*	18,307 (10)	222 (0)	<0.001
P01AB01	*Metronidazole (oral)*	18,307 (10)	222 (0)	<0.001
Adherence to NLEMI		130,078 (80)	52,802 (69)	<0.001
Adherence to WHOMLEM		126,246 (77)	51,018 (66)	<0.001
Fixed-dose combinations		29,955 (17)	24,377 (31)	<0.001
AWaRe Classification				
Access		102,530 (57)	26,407 (34)	<0.001
Watch		75,472 (42)	51,743 (66)	<0.001
Reserve		710 (0)	343 (0)	0.147

*NLEMI* National List of Essential Medicines in India, *WHOMLEM* World Health Organization Model List of Essential Medicines

The most common diagnoses for admission and operation were inguinal hernia (12%) in the TH and calculus of kidney and ureter (13%) in the NTH, followed by hyperplasia of prostate (TH, 9%; NTH, 10%, Table 3). In total, 5,733 patients (38%) were operated in the TH and 7,825 patients (54%) in the NTH. In both hospitals, all operated patients were prescribed antibiotic prophylaxis. In the TH, the most prescribed antibiotic prophylaxis was norfloxacin (19%), followed by lincomycin (15%); while in the NTH, the most prescribed antibiotic prophylaxis was ceftriaxone (24%), followed by amikacin (11%, Table 3).

**Table 3:** Most common diagnoses and their respective treatment at surgery departments in the teaching hospital (3A) and the non-teaching hospital (3B) in Ujjain, India

A						
**Teaching hospital, *n*=15,016, N (%)**						
**Most common diagnoses**		**Operated patients**	**Most prescribed antibiotics**		**Most prescribed antibiotic prophylaxis**	
**Total**		*n*=5,733 (38)			*N*=5,733 (38)	
Inguinal hernia	1,773 (12)	1,366 (9)	Total	*N*=2,665		
			Lincomycin	536	Lincomycin	431
			Cefotaxime	574	Cefotaxime	267
Hyperplasia of prostate	1,360 (9)	722 (5)	Total	*N*=1,580		
			Norfloxacin	1,055	Norfloxacin	541
			Metronidazole (oral)	91	Cefotaxime	25
Calculus of kidney and ureter	875 (6)	395 (3)	Total	*N*=1,130		
			Norfloxacin	587	Norfloxacin	249
			Metronidazole (oral)	108	Metronidazole (oral)	29
Unspecified appendicitis	507 (3)	223 (1)	Total	*N*=1,036		
			Metronidazole (IV)	237	Metronidazole (IV)	37
			Ciprofloxacin	139	Metronidazole (oral)	37
Other gastroenteritis and colitis of infectious and unspecified origin	499 (3)	10 (0)	Total	*N*=949		
			Metronidazole (oral)	298	Metronidazole (oral)	6
			Doxycycline	253	Cefotaxime	1
Haemorrhoids and perianal venous thrombosis	355 (2)	133 (1)	Total	*N*=653		
			Metronidazole (oral)	263	Doxycycline	56
			Doxycycline	220	Metronidazole (oral)	42
Fissure and fistula of anal and rectal regions	341 (2)	141 (1)	Total	*N*=624		
			Metronidazole (oral)	212	Metronidazole (oral)	41
			Doxycycline	161	Doxycycline	37
Paralytic ileus and intestinal obstruction without hernia	327 (2)	117 (1)	Total	*N*=756		
			Metronidazole (IV)	244	Metronidazole (IV)	36
			Ciprofloxacin	161	Ciprofloxacin	21
Cellulitis	309 (2)	32 (0)	Total	*N*=625		
			Cefotaxime	116	Metronidazole (IV)	8
			Metronidazole (IV)	93	Cefotaxime	5
Follicular cysts of skin and subcutaneous tissue	306 (2)	148 (1)	Total	*N*=381		
			Cefotaxime	75	Sulfamethoxazole/ trimethoprim	40
			Sulfamethoxazole/ trimethoprim	72	Lincomycin	35
B						
**Non-teaching hospital, *n*=14,499 (%), N (%)**						
**Most common diagnoses**		**Operated patients**	**Most prescribed antibiotics**		**Most prescribed antibiotic prophylaxis**	
Total		*n*=7,825 (54)			*N*=7,825 (54)	
Calculus of kidney and ureter	1,936 (13)	1526 (11)	Total	*N*=1,936		
			Ceftriaxone	451	Ceftriaxone	375
			Amikacin	302	Amikacin	238
Hyperplasia of prostate	1,420 (10)	1,031 (7)	Total	*N*=1,411		
			Amikacin	236	Amikacin	203
			Levofloxacin	228	Levofloxacin	157
Unspecified appendicitis	672 (5)	486 (3)	Total	*N*=672		
			Cefoperazone and β-lactamase inhibitor	175	Cefoperazone and β-lactamase inhibitor	149
			Metronidazole (IV)	88	Ceftriaxone	58
Inguinal hernia	539 (4)	520 (4)	Total	*N*=539		
			Ceftriaxone	218	Ceftriaxone	214
			Amikacin	89	Amikacin	84
Paralytic ileus and intestinal obstruction without hernia	460 (3)	285 (2)	Total	*N*=460		
			Piperacillin and β-lactamase inhibitor	69	Piperacillin and β-lactamase inhibitor	50
			Ceftriaxone	66	Cefoperazone and β-lactamase inhibitor	43
Acute appendicitis	371 (3)	122 (1)	Total	*N*=371		
			Metronidazole (IV)	86	Metronidazole (IV)	27
			Ceftriaxone and β-lactamase inhibitor	60	Ceftriaxone	24
Calculus of lower urinary tract	369 (3)	235 (2)	Total	*N*=369		
			Amikacin	85	Ceftriaxone	60
			Ceftriaxone	69	Amikacin	46
Other abnormal uterine and vaginal bleeding	338 (2)	210 (1)	Total	*N*=338		
			Ceftriaxone	77	Cefoperazone and β-lactamase inhibitor	62
			Cefoperazone and β-lactamase inhibitor	72	Ceftriaxone	40
Cutaneous abscess, furuncle, and carbuncle	263 (2)	238 (2)	Total	*N*=261		
			Ceftriaxone	41	Ceftriaxone	39
			Piperacillin and β-lactamase inhibitor	34	Piperacillin and β-lactamase inhibitor	32
Fever of other and unknown origin	262 (2)	5 (0)	Total	*N*=262		
			Ceftriaxone	75	Ceftriaxone	4
			Ceftriaxone and β-lactamase inhibitor	73	Moxifloxacin	1

### Trends of antibiotic prescribing over 10 years

**Fig. 1:**
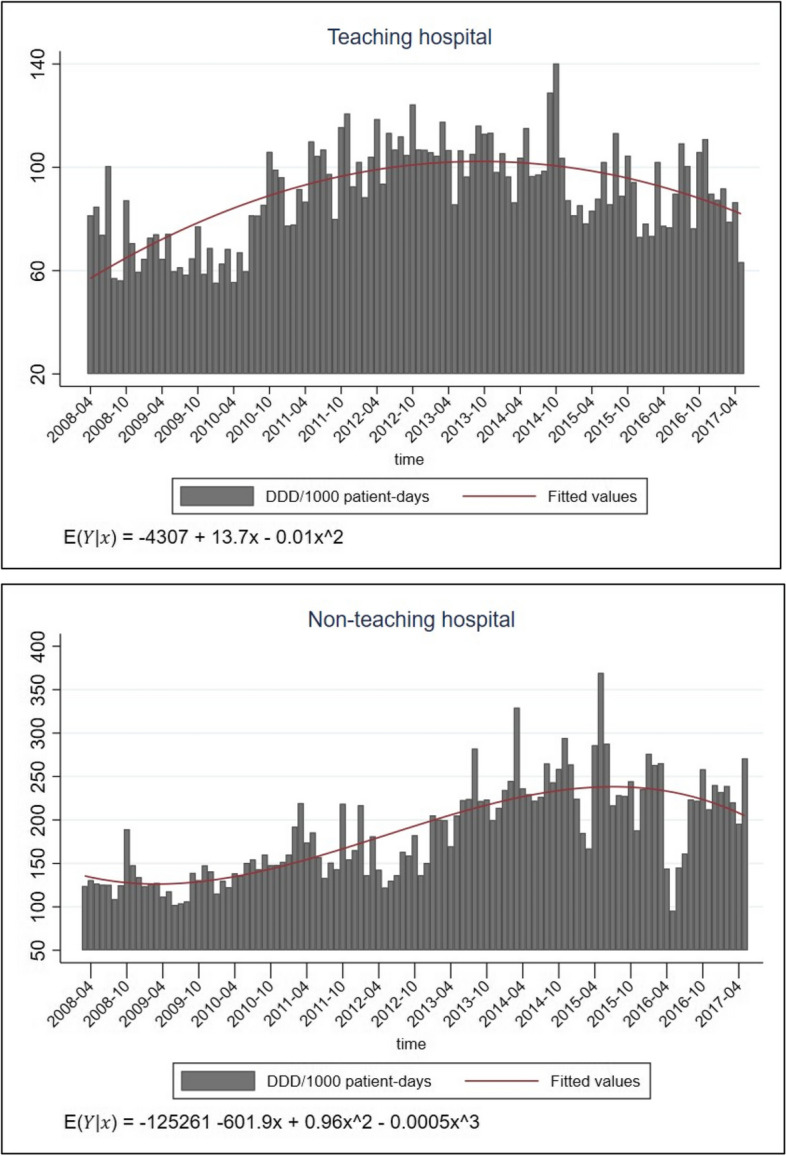
Total antibiotic consumption over 10 years at surgery departments in the teaching and the non-teaching hospital in Ujjain, India

Polynomial trends of total antibiotic consumption over 10 years in both hospitals are shown in Fig. 1. Mean DDD/1000 patient-days was two times higher in the NTH (185±56) than in the TH (90±19). In the TH, total antibiotic use was significantly increasing (β_1_ =13.7, *p*<0.001) until the year 2012, when it reached the peak (1282 DDD/1000 patient-days) and started slowly decreasing (β_2_ = -0.01, *p*<0.001). In the NTH, the total antibiotic use started slightly decreasing (β_1_ = -601.9, *p*<0.001) until the year 2009, when it started significantly increasing (β_2_= 0.96, *p*<0.001), reached the peak in the year 2014 (3049 DDD/1000 patient-days), and after it continued decreasing (β_3_= -0.0005, *p*<0.001) until 2017.

**Fig. 2:**
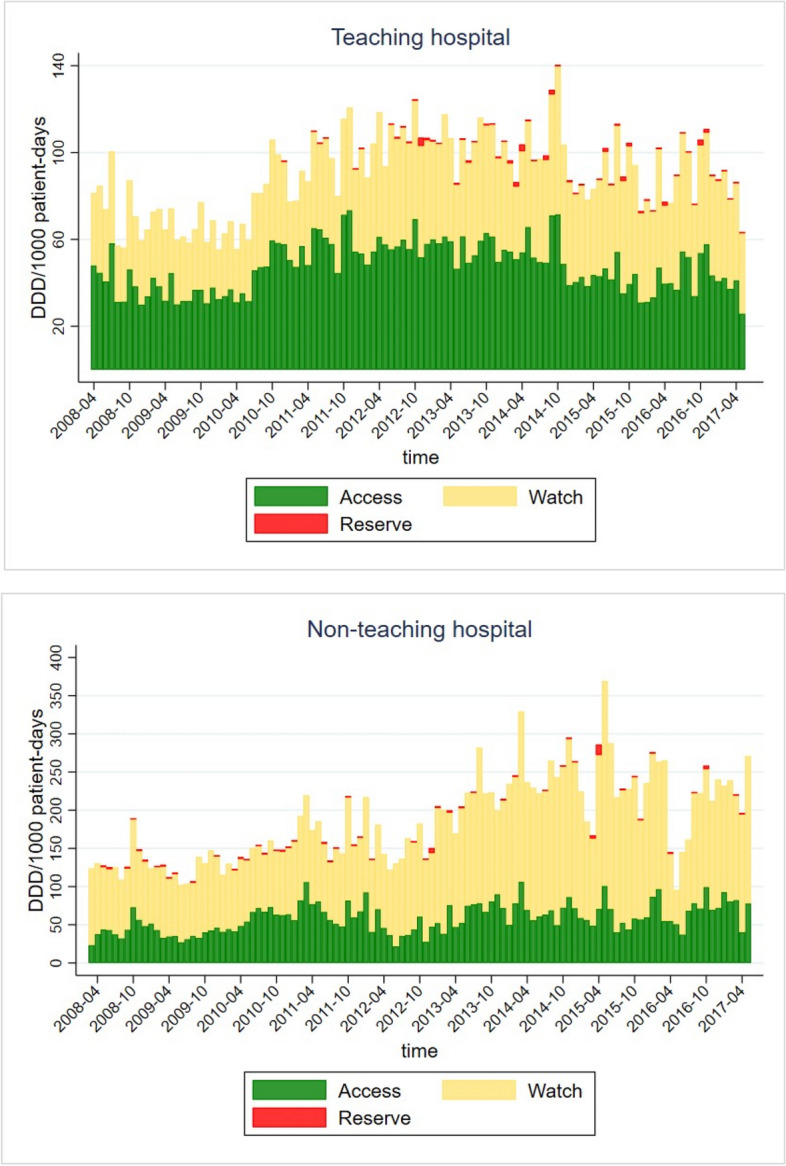
The proportion of antibiotic prescriptions per AWaRe classification over 10 years at surgery departments in the teaching and the non-teaching hospital in Ujjain, India

The change in antibiotic prescribing as per AWaRe classification over time can be seen in Fig. 2. Out of total DDD/1000 patient-days, the proportion of Access antibiotics significantly increased over 10 years in the NTH (β=0.27, *p*<0.001), while in the TH it did not significantly change (β=0.01, *p*=0.719). The proportion of Watch antibiotics significantly increased in both hospitals (TH, β=0.16, *p*<0.001; NTH, β=0.96, *p*<0.001), with the NTH having a 6 times higher rate of increase compared to the TH. The proportion of Reserve antibiotics increased slightly in the TH over 10 years (β=0.01, *p*<0.001), whereas in the NTH it did not significantly change (β=0.005, *p*=0.316, Fig. 2).

## Discussion

This was the first study to our knowledge to assess the long-term antibiotic prescribing trends at surgery departments of private-sector hospitals in Central India. Results of this study show that the most common diagnoses were inguinal hernia (TH) and calculus of the kidney and ureter (NTH). Most prescribed antibiotic classes were fluoroquinolones (TH) and 3^rd^ generation cephalosporins (NTH). All operated patients were prescribed antibiotic prophylaxis, mostly norfloxacin (TH) and ceftriaxone (NTH). In both hospitals, antibiotic prescriptions were rarely accompanied by culture and susceptibility tests, and adherence to the NLEMI was higher than adherence to the WHOMLEM. The majority of prescribed antibiotics belonged to the Access group in the TH, and the Watch group in the NTH. Total antibiotic use was two times higher in the NTH compared to the TH; showed an increasing trend until 2012 in the TH, and until 2014 in the NTH, and after that started decreasing in both hospitals. Nevertheless, the proportion of Watch antibiotic prescriptions significantly increased in both hospitals over 10 years.

All patients in this study were prescribed antibiotics even though antibiotics were largely not indicated for the observed most common diagnoses. In addition, not all patients admitted to surgery departments were operated (TH, 38%; NTH, 54%), so, the main reason for the prescription of antibiotics was not PAP. Furthermore, the most common diagnoses in both hospitals- inguinal hernia, calculus of kidney and ureter, hyperplasia of the prostate- do not classify as infectious nor high-risk for infection. PAP is not recommended for elective inguinal hernia repairs [36]. For benign hyperplasia of the prostate, the most common surgical procedure is transurethral resection of the prostate (TURP), for which PAP is indicated in all cases [37]. However, before 2015, open prostatectomy was commonly performed in the TH for the treatment of benign hyperplasia of the prostate, which is a more invasive procedure associated with higher perioperative morbidity, longer hospitalization, and prolonged recovery [38]. Thus, open prostatectomy might warrant longer postoperative antibiotic treatment, though evidence suggests that only one day of postoperative antibiotic regimen at the time of the catheter removal is sufficient [39]. In the treatment of calculus of the kidney and ureter, ureteroscopy is a common, minimally invasive procedure for which risk-adapted minimal antibiotic usage is recommended, usually in the form of a single-dose PAP [40]. For removing larger kidney and ureter stones, percutaneous nephrolithotomy is a commonly performed procedure; however, it might cause postoperative infections such as fever and urosepsis. In order to prevent postoperative infections, for patients with negative urine culture- a single dose of PAP is recommended; for patients with positive urine culture- a preoperative antibiotic treatment based on the susceptibility pattern of urine culture in duration of seven days is recommended; and for patients with positive stone culture- postoperative antibiotics based on the stone culture susceptibility pattern are recommended [41]. However, in our study, very few culture and susceptibility tests were performed, despite the functional laboratories in both hospitals. Research shows that surgical patients are more likely than other patients to receive antibiotics during their hospital stay [18]. Antibiotic prescribing without indication was reported as a frequent medical error in surgery departments [10]. Surgeons often see antibiotic prescribing as a necessary intervention, even without any evidence of infection, mainly driven by the fear of SSIs and to avoid the blame in case of complications [18, 42]. This practice was reported as the main driver of inappropriate antibiotic use in the postoperative phase [18].

In this study, all operated patients were prescribed antibiotic prophylaxis. However, PAP is not indicated for all surgeries, i.e., it is not indicated for clean, minor, and non-prosthetic-associated procedures. PAP should not be used as a substitute for adequate infection prevention and control measures and skin preparation techniques [20]. Nevertheless, surgeons in our study setting often report poor hygienic conditions of patients, as well as severe and delayed clinical presentations as the reasons to always give PAP (personal communication). The selection of PAP is based on the anatomic region to be operated, bactericidal activity, pharmacokinetic and safety profile, ease of administration of the antibiotic agent, local resistance patterns, and cost [15]. Therefore, a 1^st^ generation cephalosporin cefazolin is the recommended choice for PAP for the majority of surgical procedures unless contraindicated, as it covers the most common causative pathogens of SSIs, has good pharmacokinetic and safety profile and is cost-effective [1, 13, 15, 20]. In our study, however, a fluoroquinolone-norfloxacin (TH) and a 3^rd^ generation cephalosporin- ceftriaxone (NTH) were mostly used as antibiotic prophylaxis, and cefazolin was only prescribed in 2% of cases in both hospitals. Surgeons in the two study hospitals preferred ceftriaxone as PAP because of the good coverage of gram-positive and gram-negative bacteria in skin and soft-tissue infections and in combination with metronidazole for intra-abdominal infections [43]. Nevertheless, ceftriaxone is not recommended for all skin and soft-tissue infections, but mainly for moderate non-purulent skin and soft-tissue infections [44]. Norfloxacin was mainly given as antibiotic prophylaxis at the time of admission for urinary tract infections, which were commonly observed by the surgeons in the study setting (personal communication). As norfloxacin comes in oral formulation, it is likely that it was not given as PAP, but as antibiotic prophylaxis for urinary tract infections on the day of surgery. In addition, for a majority of surgical procedures, a single dose of intravenous PAP is adequate. Postoperative administration of antibiotics is only required in special cases, e.g. lower limb amputations and some cardiac and vascular surgeries, for a maximum duration of 24 hours [15, 20]. In our study, the median length of antibiotic therapy in both hospitals was five days and, therefore, was beyond a single dose of PAP. Extended duration of PAP is frequently reported as one of the main drivers of inappropriate antibiotic use in surgery worldwide, which increases the likelihood of adverse reactions and development of AMR [20, 26, 42].

The most prescribed antibiotic classes were fluoroquinolones in the TH and 3^rd^ generation cephalosporins in the NTH, both belonging to the Watch group of antibiotics. The predominant use of 3^rd^ generation cephalosporins and the increase in consumption of fluoroquinolones have been noted in all LMICs [4]. In India between 2011 and 2019, the amount of consumed 3^rd^ generation cephalosporins was higher than the combined amounts of 1^st^ and 2^nd^ generation cephalosporins; which resulted in 83% of bacterial isolates being resistant to 3^rd^ generation cephalosporins on the national level in 2019 [2]. Against the WHO recommendation [1], broad-spectrum 3^rd^ generation cephalosporins have long been used in India as a first choice in empiric antibiotic treatment for the respiratory tract, skin and soft tissue, and gonococcal infections, as well as for enteric fever; and have consequently become largely ineffective against infections of extended-spectrum beta-lactamase-producing (ESBL) Enterobacteriaceae [3, 4].

Further assessment of appropriateness of antibiotic prescribing at group level shows somewhat more appropriate antibiotic prescribing patterns and trends in the TH than in the NTH. In the TH, the proportion of prescribed Watch antibiotics and FDCs was lower compared to the NTH, whereas the adherences to the WHOMLEM and NLEMI were higher. Additionally, more patients were operated on and prescribed antibiotics at discharge in the NTH compared to the TH, despite the fact the hospital stay was significantly shorter in the NTH (4 days) compared to the TH (12 days). A possible explanation for this is that the TH provided services free of charge; whereas in the NTH, patients had to pay for services and medicines out-of-pocket; therefore, the expectations on surgery and antibiotic prescription both from the provider and patient side might be higher. Nevertheless, in both surgical departments, the antibiotic treatment was prolonged (5 days) and mostly empirical (≤2% culture and susceptibility tests performed), which may suggest inappropriate antibiotic use. However, in the absence of detailed information about patient comorbidities and postoperative complications of each patient, it is not possible to fully conclude on the appropriateness of antibiotic prescribing. Nonetheless, the misuse of antibiotics is a known driving force for the development of AMR [1].

In our study, total antibiotic use showed an increasing trend for the first four (TH) to six years (NTH), after which it started decreasing in both hospitals. This finding is somewhat different from the previous study conducted at the orthopaedic departments in the TH and the NTH during the same time period, which demonstrated an overall increasing trend in antibiotic use over 10 years [24]. In the study conducted by Fazaludeen Koya et al., which estimated the private-sector antibiotic consumption in India, the total antibiotic consumption, expressed by DDD/1000 inhabitants-day, decreased by 3.6% between 2011 and 2019 [2]. Furthermore, a study from Western China, also showed a decreasing trend in overall antibiotic use in hospitals between 2013 to 2015, possibly due to the National Antibiotic Stewardship Action Initiative campaign. However, the antibiotic consumption in hospitals in Western China was higher compared to the results of our study [45], keeping in mind that our study focused only on antibiotic use in surgery departments. In addition, when compared to the total antibiotic use in surgery departments in a private hospital in Indonesia (144.2 DDD/100 bed-days) [42], the results of our study showed lower mean antibiotic use in surgery departments in both hospitals. Additionally, adherence to NLEMI was higher in both hospitals (TH, 80%; NTH, 69%) compared to the reported NLEMI adherence in India (43.8%), and compared to the reported NLEMI adherence in Madhya Pradesh (44%) [2]. Furthermore, the proportion of prescribed FDCs was lower in both hospitals (TH, 17%; NTH, 31%) compared to the relatively high proportion of FDC prescriptions in Madhya Pradesh (41.2%), which is among the highest in India [2].

The WHO target of a minimum of 60% Access antibiotics of all prescribed antibiotics [1] was not met in our study. Overall, the TH had 57% of Access antibiotic prescriptions compared to only 34% in the NTH. The proportion of Access antibiotics is higher in the TH and lower in the NTH compared to the reported Access/Watch ratio (0.5) in the private sector in Madhya Pradesh between 2011 and 2019 [2]. In our study, the proportion of Access antibiotics increased in the NTH between 2008 and 2017, while in the TH it did not change significantly. The proportion of Watch antibiotics increased in both hospitals; whereas the proportion of Reserve antibiotics increased slightly in the TH, while in the NTH it did not change significantly. In comparison, when looking into the private sector consumption in India between 2011 and 2019, the reported consumption of Access antibiotics decreased by 13%, of Watch antibiotics increased by 4%, and of Reserve antibiotics increased by 247% [2]. Although direct comparison of levels of change is difficult between the two studies due to the different methods of analysis used, we can observe that the trend of increase in consumption of Watch antibiotics in our study is consistent with the overall trend in India. Furthermore, the proportion of consumed Reserve antibiotics between 2011 and 2019 in India remained approximately 1% [2], which is similar to the proportion of consumed Reserve antibiotics in our study (<1%). It has to be noted, however, that the WHO target of 60% of Access antibiotics refers to the overall antibiotic use at a country level [1], whereas our study setting is limited to two tertiary-care hospitals, and the use of Watch antibiotics is expected to be higher in the tertiary-care settings compared to the primary-care settings.

One of the biggest strengths of the present study is the long duration and a large sample size, which can be considered representative for the surgical patients in Central India. The data collection process was prospective, and the same data collection tool was used in both hospitals, training and monitoring was done by the same person to maintain the uniformity and enable the comparison. The study used the ATC/DDD system, which is the recommended method for the comparison of antibiotic use between healthcare settings. However, this method gives only one DDD per drug, while the recommended dose can differ based on age, type, and severity of diagnosis [32]. Additionally, DDDs can change over time, which can complicate the comparison of trends in antibiotic use over longer periods of time. Furthermore, although the latest versions of essential medicines lists (2022 NLEMI, 2023 WHOMLEM) were not applicable at the time of the study, they were used alongside AWaRe classification for the analysis of antibiotic prescribing to make it more relevant to present-day and comparable with the current recommendations.

One of the limitations of the manual data collection was the possibility of missing and incorrectly entered data. However, the utilization of nurses working in the department, for data collection is a novel concept which can be replicated at other similar settings that do not have computerized data entry system. The data collection form was not designed to collect information about PAP exclusively, therefore, antibiotic prescribed on the day of surgery was considered as PAP for analysis, that can be an underestimation. In addition, information about the culture and susceptibility tests started to be collected from 2011, approximately three years after the beginning of the study, therefore, it is possibly lowballed. Additionally, the diagnoses in this study were analysed at a category level, whereas analysis of the individual diagnoses, as well as more detailed information about the type of wound and surgery, associated patient and surgical risk factors, skin preparation, timing and duration of PAP, and postoperative complications would have been necessary to fully assess the appropriateness of antibiotic prescribing. Therefore, the appropriateness of antibiotic prescribing could only be partially assessed in this study.

### Public health implications

#### Clinical practice recommendations

- Development and implementation of antibiotic prescribing and PAP guidelines in surgery departments based on local resistance patterns.
- Performance of culture and susceptibility tests when appropriate to establish local resistance patterns and AMR burden in the study setting.

#### Future research

Identification of the underlying reasons for the observed antibiotic prescribing practices, the understanding of which will help in the design of targeted interventions.

#### Antimicrobial stewardship program policy

- Development and implementation of multidisciplinary antimicrobial stewardship programs in both hospitals, as they demonstrated a significant improvement in antibiotic prescribing practices and reduction of healthcare-associated infections [20, 46].
- Active surveillance in the form of frequent audits and feedback to all healthcare staff involved in perioperative and postoperative care, to improve patient outcomes and reduce healthcare costs and AMR [17].

## Conclusion

Total antibiotic prescribing increased until 2012 in the TH and 2014 in the NTH, after which it started decreasing in both hospitals until 2017. Consumption of Watch antibiotics increased over 10 years in both hospitals. Antibiotic prophylaxis was prescribed to all operated patients, although it is not indicated for every surgery, and the observed most common diagnoses were not high-risk nor infectious. Most prescribed antibiotic prophylaxes were norfloxacin and ceftriaxone, contrary to the internationally recommended PAP standard cefazolin. Antibiotic prescriptions were largely empirical. The results of this study suggest the need to perform culture and susceptibility tests whenever appropriate in order to establish local resistance patterns. There is a pressing need for the development and implementation of antibiotic prescribing and PAP guidelines in surgery based on local resistance patterns, as well as antimicrobial stewardship programs in both hospitals.

## Data Availability

It is not possible to share research data publicly due to the threat that individual privacy could be compromised. Therefore, restrictions apply to the availability of these data. The data that support the findings of this study are, however, available upon reasonable request to the authors and with permission of the Chairman of the Ethics Committee, R.D. Gardi Medical College, Agar Road, Ujjain; stating the reference number and reason for asking for access.

## Abbreviations

AMR: Antimicrobial resistance
ATC: Anatomical therapeutic chemical
AWaRe: Access Watch Reserve
DDD: Defined daily dose
ESBLs: Extended-spectrum beta-lactamases
FDC: Fixed-dose combination
ICD: International statistical classification of diseases and related health problems
LMIC: Low- and middle-income country
NLEMI: National List of Essential Medicines India
NTH: Non-teaching hospital
PAP: Perioperative antibiotic prophylaxis
SSI: Surgical site infection
TH: Teaching hospital
TURP: Transurethral resection of the prostate
WHO: World Health Organization
WHOCC: World Health Organization Collaborating Centre for Drug Statistics Methodology
WHOMLEM: World Health Organization Model List of Essential Medicines

## References

[1] World Health Organization. The WHO AWaRe (Access, Watch, Reserve) antibiotic book. Geneva: World Health Organization; 2022. Cited 2023 Jan 9. Available from: https://www.who.int/publications/i/item/9789240062382.

[2] Fazaludeen Koya S, Ganesh S, Selvaraj S, Wirtz VJ, Galea S, Rockers PC. Antibiotic consumption in India: geographical variations and temporal changes between 2011 and 2019. JAC-Antimicrobial Resistance. 2022;4(5):dlac112. 10.1093/jacamr/dlac112 PMC9596537 36320447

[3] Gandra S, Tseng KK, Arora A, Bhowmik B, Robinson ML, Panigrahi B (2019). The mortality burden of multidrug-resistant pathogens in India: a retrospective observational study. Clin Infect Dis.

[4] Klein EY, Van Boeckel TP, Martinez EM, Pant S, Gandra S, Levin SA, et al. Global increase and geographic convergence in antibiotic consumption between 2000 and 2015. Proc Natl Acad Sci USA. 2018 ;115(15). Cited 2023 Jan 21. Available from: https://pnas.org/doi/full/10.1073/pnas.1717295115. 10.1073/pnas.1717295115 PMC5899442 29581252

[5] Pokharel S, Raut S, Adhikari B (2019). Tackling antimicrobial resistance in low-income and middle-income countries. BMJ Glob Health.

[6] Dixit A, Kumar N, Kumar S, Trigun V (2019). Antimicrobial resistance: progress in the decade since emergence of new delhi metallo-β-Lactamase in India. Indian J Community Med.

[7] Klein EY, Tseng KK, Pant S, Laxminarayan R (2019). Tracking global trends in the effectiveness of antibiotic therapy using the Drug Resistance Index. BMJ Glob Health.

[8] Monahan M, Jowett S, Pinkney T, Brocklehurst P, Morton DG, Abdali Z, et al. Surgical site infection and costs in low- and middle-income countries: A systematic review of the economic burden. PLoS One. 2020;15(6):e0232960. Serra R, editor. 10.1371/journal.pone.0232960 PMC7272045 32497086

[9] Mezemir R, Seid A, Gishu T, Demas T, Gize A (2020). Prevalence and root causes of surgical site infections at an academic trauma and burn center in Ethiopia: a cross-sectional study. Patient Saf Surg.

[10] Cusini A, Rampini SK, Bansal V, Ledergerber B, Kuster SP, Ruef C (2010). Different Patterns of Inappropriate Antimicrobial Use in Surgical and Medical Units at a Tertiary Care Hospital in Switzerland: A Prevalence Survey. PLoS One.

[11] European Centre for Disease Prevention and Control. Surveillance of surgical site infections and prevention indicators in European hospitals: HAI Net SSI protocol, version 2.2. LU: Publications Office; 2017. Cited 2023 Apr 15. Available from: https://data.europa.eu/doi/10.2900/260119.

[12] European Centre for Disease Prevention and Control. Healthcare-associated infections: surgical site infections. Annual epidemiological report for 2017. Stockholm: ECDC; 2019. Cited 2023 May 21. Available from: https://www.ecdc.europa.eu/sites/default/files/documents/AER_for_2017-SSI.pdf.

[13] World Health Organization. Global guidelines for the prevention of surgical site infection. Geneva: World Health Organization; 2016. 184. Cited 2023 Jul 24. Available from: https://apps.who.int/iris/handle/10665/250680.

[14] ECDC., Public Health England., Institut de Veillle Sanitaire. Systematic review and evidence-based guidance on perioperative antibiotic prophylaxis. Stockholm, Sweden: Publications Office; 2013. Cited 2023 Jan 9. Available from: https://data.europa.eu/doi/10.2900/85936.

[15] Crader M, Varacallo M. Preoperative Antibiotic Prophylaxis. Treasure Island (FL): StatPearls; 2023. Cited 2023 Jul 24. Available from: https://www.ncbi.nlm.nih.gov/books/NBK442032/. 28723061

[16] Ratnesh K, Kumar P, Arya A (2022). Incidence of Surgical Site Infections and Surgical Antimicrobial Prophylaxis in JNMC, Bhagalpur, India. J Pharm Bioall Sci.

[17] Ahuja S, Peiffer-Smadja N, Peven K, White M, Leather AJM, Singh S (2022). Use of feedback data to reduce surgical site infections and optimize antibiotic use in surgery: a systematic scoping review. Ann Surg.

[18] Charani E, Tarrant C, Moorthy K, Sevdalis N, Brennan L, Holmes AH (2017). Understanding antibiotic decision making in surgery—a qualitative analysis. Clin Microbiol Infect.

[19] Australian Commission on Safety and Quality in Health Care. Antimicrobial Prescribing Practice in Australian Hospital: Results of the 2018 Hospital National Antimicrobial Prescribing Survey. 2020. Cited 2023 Jul 28. Available from: https://www.safetyandquality.gov.au/sites/default/files/2020-02/report_-_2018_hospital_naps.pdf.

[20] Ierano C, Manski-Nankervis JA, James R, Rajkhowa A, Peel T, Thursky K (2017). Surgical antimicrobial prophylaxis. Aust Prescr.

[21] Allegranzi B, Nejad SB, Combescure C, Graafmans W, Attar H, Donaldson L (2011). Burden of endemic health-care-associated infection in developing countries: systematic review and meta-analysis. Lancet.

[22] Mekhla, Borle F. Determinants of superficial surgical site infections in abdominal surgeries at a Rural Teaching Hospital in Central India: a prospective study. J Fam Med Prim Care. 2019;8(7):2258. 10.4103/jfmpc.jfmpc_419_19 PMC6691442 31463239

[23] Lindsjö C, Sharma M, Mahadik VK, Sharma S, Stålsby Lundborg C, Pathak A (2015). Surgical site infections, occurrence, and risk factors, before and after an alcohol-based handrub intervention in a general surgical department in a rural hospital in Ujjain India. Am J Infect Control.

[24] Skender K, Machowska A, Singh V, Goel V, Marothi Y, Lundborg CS (2022). Antibiotic use, incidence and risk factors for orthopedic surgical site infections in a teaching hospital in Madhya Pradesh, India. Antibiotics.

[25] Sparrentoft J, Sharma M. Perioperative antibiotic prescribing in two private sector hospitals in central India. Antimicrob Resist Infect Control. 2015;4(S1):P176, 2047-2994-4-S1-P176.

[26] Santoshi JA, Behera P, Nagar M, Sen R, Chatterjee A (2021). Current surgical antibiotic prophylaxis practices: a survey of orthopaedic surgeons in India. IJOO.

[27] Swain S, Preetha G, Kumar S, Aggarwal D, Kumar R, Kumar S (2020). Human resources for health in India: need to go beyond numbers. Indian J Community Med.

[28] Machowska A, Sparrentoft J, Dhakaita SK, StålsbyLundborg C, Sharma M (2019). Perioperative antibiotic prescribing in surgery departments of two private sector hospitals in Madhya Pradesh, India. Perioper Med..

[29] Sharma M, Damlin AL, Sharma A, Stålsby Lundborg C (2015). Antibiotic prescribing in medical intensive care units – a comparison between two private sector hospitals in Central India. Infect Dis.

[30] Sharma M, Eriksson B, Marrone G, Dhaneria S, Lundborg CS (2012). Antibiotic prescribing in two private sector hospitals; one teaching and one non-teaching: a cross-sectional study in Ujjain, India. BMC Infect Dis.

[31] Skender K, Singh V, Stalsby-Lundborg C, Sharma M (2021). Trends and patterns of antibiotic prescribing at orthopedic inpatient departments of two private-sector hospitals in Central India: A 10-year observational study. PLoS One.

[32] World Health Organization, Collaborating Centre for Drug Statistics Methodology. ATC classification index with DDDs. WHO Collaborating Centre for Drug Statistics Methodology. Cited 2023 Apr 15. Available from: https://www.whocc.no/use_of_atc_ddd/.

[33] The Core-Committee. National List of Essential Medicines (NLEM) 2022. The Government of India, Ministry of Health and Family Welfare (MOHFW); 2022. Cited 2023 May 22. Available from: https://main.mohfw.gov.in/newshighlights-104.

[34] World Health Organization. World Health Organization Model List of Essential Medicines – 23rd List, 2023. World Health Organization; 2023. Cited 2023 Aug 22. Available from: https://www.who.int/publications/i/item/WHO-MHP-HPS-EML-2023.02.

[35] World Health Organization. International Statistical Classification of Disease and Related Health Problems - 10th revision. 2016. World Health Organization; 2016. Cited 2023 Jan 22. Available from: https://icd.who.int/browse10/2016/en.

[36] Zamkowski MT, Makarewicz W, Ropel J, Bobowicz M, Kąkol M, Śmietański M. Antibiotic prophylaxis in open inguinal hernia repair: a literature review and summary of current knowledge. wiitm. 2016;3:127–36. 10.5114/wiitm.2016.62800 PMC5095278 27829934

[37] Lawson KA, Rudzinski JK, Vicas I, Carlson KV (2013). Assessment of antibiotic prophylaxis prescribing patterns for TURP: a need for Canadian Guidelines?. CUAJ.

[38] Lin Y, Wu X, Xu A, Ren R, Zhou X, Wen Y (2016). Transurethral enucleation of the prostate versus transvesical open prostatectomy for large benign prostatic hyperplasia: a systematic review and meta-analysis of randomized controlled trials. World J Urol.

[39] Ehdaie B, Jibara G, Sjoberg DD, Laudone V, Eastham J, Touijer K (2021). The duration of antibiotics prophylaxis at the time of catheter removal after radical prostatectomy: clinically integrated, cluster, randomized trial. J Urol.

[40] Schnabel MJ, Wagenlehner FME, Schneidewind L (2019). Perioperative antibiotic prophylaxis for stone therapy. Curr Opin Urol.

[41] Zeng T, Chen D, Wu W, Huang Y, Zhang S, Zhao Z (2020). Optimal perioperative antibiotic strategy for kidney stone patients treated with percutaneous nephrolithotomy. Int J Infect Dis.

[42] Muliani N, Herawati F, Yulia R, Wijono H (2021). Quantity and quality profiles of antibiotics pre, on, and post surgery in a hospital setting. Int J Clin Pharm.

[43] Tan A, Rouse M, Kew N, Qin S, La Paglia D, Pham T (2018). The appropriateness of ceftriaxone and metronidazole as empirical therapy in managing complicated intra-abdominal infection—experience from Western Health, Australia. PeerJ.

[44] Stevens DL, Bisno AL, Chambers HF, Dellinger EP, Goldstein EJC, Gorbach SL (2014). Executive summary: practice guidelines for the diagnosis and management of skin and soft tissue infections: 2014 update by the infectious diseases society of America. Clin Infect Dis.

[45] Yan K, Xue M, Ye D, Yang C, Chang J, Jiang M (2018). Antibiotic prescribing practices in secondary and tertiary hospitals in Shaanxi province, western China, 2013–2015. PLoS One.

[46] World Health Organization. Antimicrobial stewardship programmes in health-care facilities in low- and middle-income countries: a WHO practical toolkit. Geneva: World Health Organization; 2019. 71. Cited 2023 Jul 25. Available from: https://apps.who.int/iris/handle/10665/329404.

